# The impact of conscientious objection on voluntary abortion in Italy in the last two decades

**DOI:** 10.1093/eurpub/ckac129.670

**Published:** 2022-10-25

**Authors:** L Charrier, M Bo, E Koumantakis, CM Zotti

**Affiliations:** 1 Public Health and Pediatrics, University of Torino, Turin, Italy; 2 Hospital Medical Direction, Local Health Authority Torino 5, Chieri, Italy

## Abstract

**Background:**

Italian Law 194/1978 legalized voluntary abortion during the first 90 days of pregnancy. Healthcare professionals can claim conscientious objection (CO), but regional governments must guarantee women's rights. In recent years, international human rights authorities argued that access to safe abortion was limited in Italy due to widespread CO.

**Methods:**

An ecological study was conducted using 1997-2019 ministerial data on the number of gynecologists registered as conscientious objectors, the proportion of abortions performed timely (within 14 days of the request), and within 8-10 weeks of pregnancy. The extra workload for non-objecting gynecologists was calculated as the ratio between the workload for non-objectors and the (theoretical) workload for each gynecologist on duty. The correlation between the extra workload for non-objectors and the proportion of abortions performed within 14 days of the request or beyond 21, as well as the correlation between waiting time and gestational age at the time of the procedure. Data were analyzed for Italy and stratified for its 21 Regions.

**Results:**

CO among gynecologists turned out to be stable around an average of 70% (median 69%, IQR 64-71%) in the last decades, with 62% of abortions performed within 14 days and 82% of procedures performed by the 10th week of pregnancy. In 13 regions (statistically significant in 5) the increase in workload for non-objectors was inversely correlated with abortions performed within 14 days, and directly correlated with those performed later than 21 days. In all regions (statistically significant in 18) a direct correlation was found between procedures performed timely and those performed within 8 weeks of pregnancy.

**Conclusions:**

Data from the last 20 years confirm previous findings and CO still seems to have a strong impact on women's right to access safe and timely abortion in Italy. More efforts are needed to narrow the gap between the provisions of the law and its implementation.

**Key messages:**

• A high proportion of objecting staff makes it difficult to guarantee women’s rights to access timely and safe abortion.

• Effective organizational strategies and a proper legal framework are needed to cope with the high percentage of conscientious objectors among health professionals.

